# Supplementary material for: Motor planning flexibly optimizes performance under uncertainty about task goals
Source: Nat Commun. 2017 Mar 3;8:14624. doi: 10.1038/ncomms14624 (PMC5337982; doi:10.1038/ncomms14624)
Supplement: Supplementary Information — Supplementary Figure and Supplementary Table. [file ncomms14624-s1.pdf]

Supplementary Figure 1. Reaching trajectories from all participants in Experiment 1.

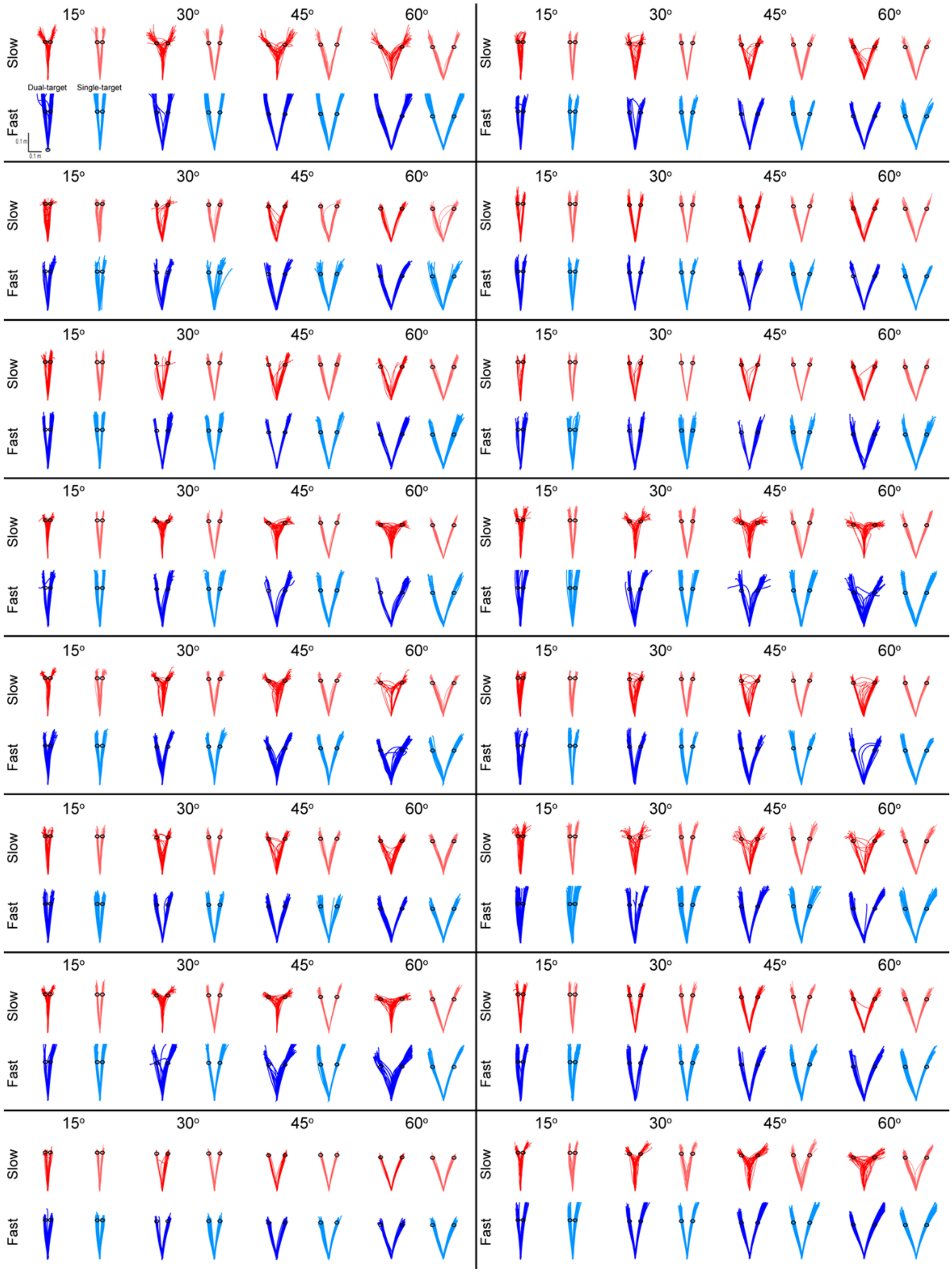

Reaches are presented from the 16 participants included in Experiment 1. Reaches are grouped by target-separation angle (horizontal) and required movement speed (vertical; Slow reaches in red, Fast reaches in blue). Both dual-target trials (darker colors) and single-target trials (lighter colors) are shown. These data highlight both the general group-wide tendency to produce intermediate movements mainly for Slow trials. Additionally, it is possible to observe the participants who generated no intermediate movements throughout the entire session.

Supplementary Table 1. Group-wide model parameters.

Target Separation	Fast					Slow			
	μ_d	σ_d	σ_i	θ		μ_d	σ_d	σ_i	θ
15°	7.562	3.046	8.238	0.078		5.052	4.242	5.810	0.500
30°	15.359	3.210	4.816	0.071		13.149	4.672	3.031	0.329
45°	22.205	3.353	4.884	0.045		19.964	4.964	3.836	0.397
60°	29.058	3.382	18.960	0.098		26.074	5.193	4.934	0.352

Parameters shown above reflect the model fits to the pooled data (stage-1 fits), fitted to the data from Experiment 1. The particularly large estimate of σ_i for in the Fast 60° condition reflects the fact that it was very rare to observe intermediate movements in this condition.
